# Rapid Communication: Plasma Interleukin-35 in Children with Autism

**DOI:** 10.3390/brainsci9070152

**Published:** 2019-06-27

**Authors:** Destanie Rose, Paul Ashwood

**Affiliations:** Department of Medical Microbiology and Immunology, and The Medical Investigation of Neurodevelopmental Disorders Institute, University of California, Davis, CA 95817, USA

**Keywords:** autism spectrum disorders, cytokines, immune regulation, interleukin, IL-35, anti-inflammatory

## Abstract

In autism spectrum disorders (ASD) many individuals have co-morbid immune dysregulation that can lead to inflammation in the brain and periphery. The novel cytokine interleukin (IL)-35 has described anti-inflammatory properties; however, the plasma levels of IL-35 in children with ASD have never been investigated. The plasma levels of IL-35 were measured by an enzyme-linked immunosorbent assay in 30 children with ASD and 39 typically developing (TD) controls. In the current study, we found that plasma IL-35 levels were significantly decreased in children with ASD compared with TD children. Furthermore, lower IL-35 levels were associated with worse behaviors as assessed using the aberrant behavior checklist. These findings are in line with other observations of decreased regulatory cytokines such as transforming growth factor beta and IL-10 in ASD, and associations with severity of behaviors. In conclusion, regulating the expression of IL-35 may provide a new possible target for the treatment of immune issues in ASD to address an imbalance between pro- and anti-inflammatory signals that alter the behavioral phenotype.

## 1. Introduction

The prevalence of autism spectrum disorders (ASD) has seen dramatic rises over the last 20 years and is now estimated to affect nearly 2% of young children in the United States [1]. Defined by behavioral criteria based on impairments in social interactions, communication, and repetitive behaviors, the etiology of ASD is still largely unknown but likely involves complex environmental and genetic factors [2,3,4]. Aberrant immune responses have been postulated to be involved in ASD pathophysiology, either as a cause of autism-like symptoms (as highlighted in animal models and human studies that involve maternal immune activation), or through ongoing immune activation which can lead to more impaired behaviors [5,6,7,8,9]. Increased allergies, asthma and autoimmunity have also been shown in ASD [10,11,12,13,14,15,16]. In many studies, the focus has been on the activation or increase of pro-inflammatory elements such as cytokines, T cells, innate immune cells, and auto-antibodies (reviewed in Hughes [2]). While compelling evidence highlights that multiple effectors are activated in ASD, no clear consensus has been reached as to a specific response mechanism with many different arms of the immune response being activated [17]. In fact, recent data suggests that an underlying mechanism may be the lack of immune regulation.

Immune regulation is mediated by the combination of regulatory immune cells and the release of immune suppressive or anti-inflammatory cytokines. In ASD, several studies have shown that regulatory cytokines such as transforming growth factor beta-1 (TGFβ1) or interleukin (IL)-10 are decreased [9,18,19,20,21,22,23,24,25]). Moreover, decreases in regulatory T cells (T_regs_) have also been described [26,27]. 

IL-35 is a novel cytokine with recently described immune suppressive activities. It is a member of the IL-12 family of cytokines composed of an alpha chain (p35) and a beta chain that is a L-27 subunit Epstein–Barr virus-induced gene 3 (EBI3). IL-35 is released by regulatory CD4^+^ and CD8^+^ T (T_reg_) cells, dendritic cells, and regulatory B cells. The main effector mechanism of IL-35 appears in the inhibition of T helper type 17 (T_H_17) and proliferation of T_reg_. Decreased IL-35 has been shown in auto-inflammatory conditions, such as multiple sclerosis, inflammatory bowel disease, systemic lupus erythematosus, rheumatoid arthritis, and psoriasis [28,29]. 

We have previously hypothesized that there is a dysregulation in the balance between T_H_17 and T_reg_ cells in ASD [9], thus potentially making IL-35 a key player in this dynamic. In this pilot study, we sought to examine plasma levels of IL-35 in children with ASD and age-, and geographically matched control samples from typically developing children. In addition, we examined whether there were associations between IL-35 levels and subscale scores on the Aberrant Behavior Checklist (ABC).

## 2. Methods

### 2.1. Subjects

The recruitment of study participants was through the UC Davis MIND Institute. Each participant was previously enrolled in the Childhood Autism Risk from Genetics and Environment (CHARGE) study [30]. A total of 69 participants comprised this study, composed of 30 children with ASD (median: 7.7 years of age; interquartile range (IQR): 5.3–10.3 years; 26 males, 4 females) and 39 who were typically developing (TD) (7 years of age; 5.6–8.4 years; 36 males, 3 females). The participants’ age ranged from 3 to 12 years old. The autism spectrum disorder diagnosis was verified by trained clinicians at the UC Davis MIND Institute and the diagnoses occurred before 2013 and were, therefore, based on DSM IV following the Autism Diagnostic Observation Schedule (ADOS) and the Autism Diagnostic Interview-Revised (ADI-R). The diagnosis was determined between the age of 2–5 when the participants were originally enrolled into the CHARGE study. Each participant who was placed in the control group was prescreened with the Social Communication Questionnaire (SCQ), the Mullen Scales of Early Learning (MSEL), and the Vineland Adaptive Behavior Score (VABS) in order to eliminate behavioral and developmental characteristics of ASD. TD participants were required to score inside the typical range, specifically below 15, on the SCQ and above 70 on the MSEL and VABS. The subjects for this study were recruited at random from the CHARGE study database, care was taken to ensure typically developing subjects were frequency matched to ASD subjects based on birth location, age, and sex. All subjects were evaluated using the Aberrant Behavior Checklist (ABC) to evaluate impairments within the domains of lethargy, stereotypic behavior, social withdrawal, irritability, hyperactivity, and inappropriate speech when they were enrolled in the current study. Medications that were used at the time of enrollment were recorded and they included Albuterol, Claritin, Cimetidine, Clonidine, Flonase, Flovent, Fluticasone, Guanfacine, Ibuprofen, Qvar, Risperidone, Singulair, Strattera, Tenex, Triaminic, Tylenol, Zantac, and Zyrtec. Excluding the 12 subjects who were taking one or more of the listed medications did not significantly alter the results so they were included in the final analysis presented here. 

Participants were excluded if they met any of these exclusion criteria: evidence of fever, genetic disorders (such as Fragile X syndrome or Tuberous Sclerosis Complex), seizure disorder, pancreatic or liver disease, cystic fibrosis, or other chronic infections. 

The approval for this study was given by institutional review boards for the State of California and the University of California, Davis. In accordance with UC Davis IRB protocol, informed consent was obtained from a legal guardian for all study participants prior to data collection. 

### 2.2. Blood Collection and Cytokine Analysis

Peripheral blood was obtained from each participant in acid-citrate dextrose Vacutainers (BD Biosciences; San Jose, CA, USA) and processed within 8 hours of being drawn. Vacutainers containing blood were centrifuged at a speed of 2100 rpm for 10 minutes, plasma was collected, aliquoted, and stored at −80° until analysis.

The quantification of IL-35 was completed via a multiplexing bead immunoassay (Millipore, Billerica, MA, USA) following the manufacturer’s protocol. Briefly, overnight incubation of 25 µL of 1:2 diluted plasma with antibody coupled fluorescent beads, was followed by a washing step before further incubation with biotinylated detection antibodies for 1 hour preceding the addition and incubation of streptavidin–phycoerythrin for 30 min before one final wash. Following the last wash the beads were analyzed on the flow-based Luminex™ 100 suspension array system (Bio-Plex 200; Bio-Rad Laboratories, Inc., Hercules, CA, USA). To determine unknown sample concentrations, a standard curve was prepared using reference IL-35 provided by the manufacturer in the kit and ran in the same assay with the samples. The kit listed the minimum detectable amount of IL-35 as 0.3 ng/mL. Samples with concentrations that were determined to be lower than the limit of detection were given a proxy value of half the limit of detection for statistical comparisons. 

### 2.3. Statistical Analysis

Performing a Shapiro–Wilk normality test on the IL-35 data revealed non-parametric distribution of the data, in which standard transformation processes (i.e., log transformation, square-root transformation) could not normalize, therefore, the data is presented as median values with interquartile ranges in parentheses. A Mann–Whitney non-parametric *U*-test was used to compare IL-35 concentrations across study groups. Findings were considered significant if adjusted *p* values were less than 0.05. Outliers were eliminated using ROUT. Associations between behavioral data (ABC subscales) and plasma IL-35 concentrations were analyzed utilizing the non-parametric, Spearman’s correlation. 

## 3. Results

**Figure 1:**
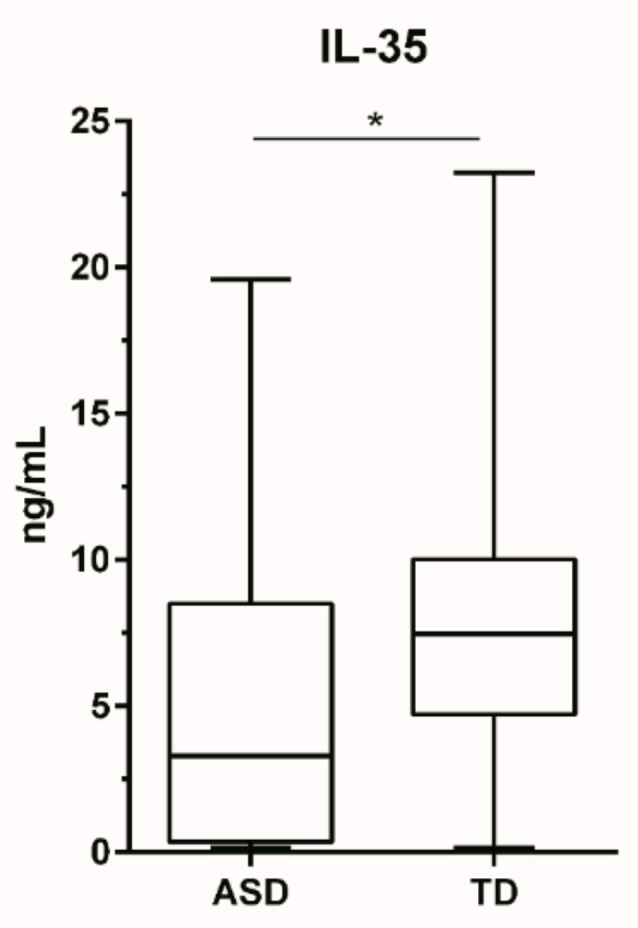
Comparison of plasma IL-35 levels (ng/mL) in children with autism (*n* = 30) and typically developing controls (*n* = 39). Data is depicted as box and whisker graphs, comparing medians. * denotes *p*-value > 0.05.

**Figure 2:**
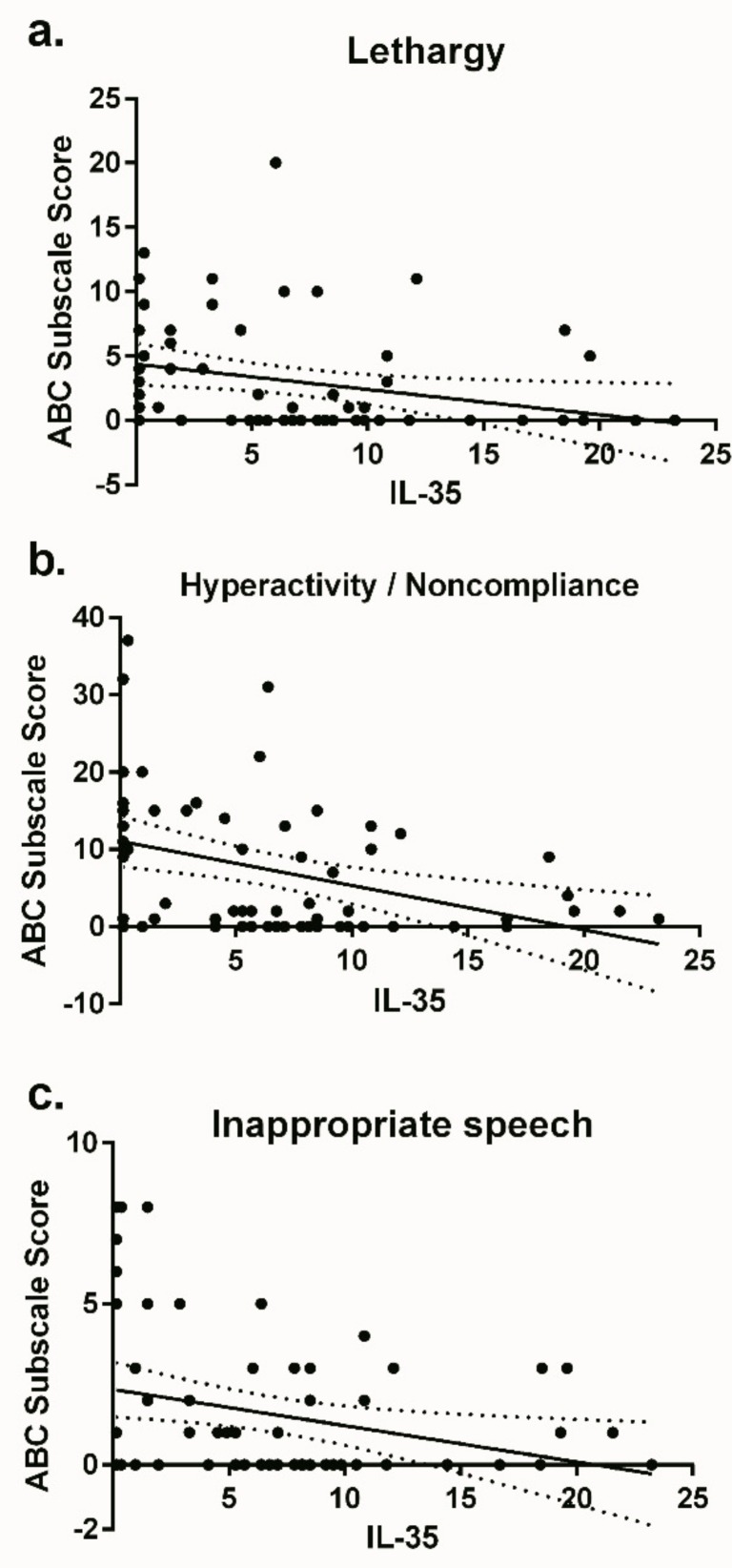
Plasma IL-35 concentration correlated behavioral data from the ABC subscales, lethargy (**a**), hyperactivity/noncompliance (**b**), and inappropriate speech (**c**). Data presented as scatter plots with linear regression lines and 95% confidence lines.

Plasma from children with ASD contained lower concentrations of the regulatory cytokine IL-35 (median: 3.30 ng/mL; IQR: 0.35–8.51 ng/mL) compared to typically developing children (7.47 ng/mL; 4.71–10.02 ng/mL) *p* = 0.023 (Figure 1). When analyzing for associations with behavioral data using ABC assessments, we found that plasma IL-35 concentrations were inversely correlated with lethargy (*r*: −0.388; *p* = 0.002), hyperactivity (*r*: −0.368; *p* = 0.003), and inappropriate speech (*r*: −0.250; *p* = 0.046) (Figure 2).

## 4. Discussion

To the best of our knowledge, this is the first study to assess the plasma IL-35 concentration in ASD. Our results demonstrated that there are decreased levels of plasma IL-35 in children with ASD. Moreover, we found associations between IL-35 levels and more impairment in behavior. It is currently unknown whether IL-35 can affect neuronal function, neurodevelopment and consequently directly alter behaviors, and this data should be treated with caution. However, of note, multiple studies have shown that impairments in core ASD features and associated behaviors, are strongly correlated with altered immune profiles [2,3,4,31]. A decrease in immune regulation via IL-35 could lead to a tip in the balance towards enhanced immune activation that could alter neurodevelopment. Further validation of the link between observed behavioral severity and IL-35 its receptors/signaling is warranted in ASD.

Decreased levels of anti-inflammatory immunosuppressive cytokines, such as TGFβ1 and IL-10 have previously been shown in ASD. We and others have shown that plasma levels of TGFβ1 were reduced in ASD compared to TD controls and children with other developmental disabilities [18,22,32]. Lower TGFβ1 levels in children with ASD were associated with fewer social interactions, worse adaptive behaviors, hyperactivity, irritability, and stereotypies [18]. Furthermore, in the analysis of 414 ASD and 820 TD newborn blood spots, TGFβ1 levels were significantly decreased in ASD [33]. Adding further support to decreased regulation playing a role in the pathophysiology of ASD, differentially expressed microRNAs (miRNAs) that control TGFβ1 pathways were found in ASD [34,35,36]. In cell stimulation assays, studies showed that the production of IL-10 by CD4^+^ T cells was reduced in ASD [20,21,37,38]. Peripheral blood mononuclear cells (PBMC) also produce less TGFβ1 upon stimulation [9]. These data suggest there is a lack of immune regulation due to decreased production of one or more regulatory cytokine in children with ASD and is associated with worse behavior.

Regulatory T cells are also decreased in ASD. CD4^+^CD25^++^ cells are considered enriched for a population of cells with suppressive activity. We assessed CD4^+^CD25^++^ T cells by flow cytometry in 65 children with a confirmed diagnosis of ASD and 73 TD controls and found an ~30% decrease in the frequency of CD4^+^CD25^++^ cells in ASD [26]. The circulating frequency of CD4^+^CD25^+^ T_regs_ in blood was also examined in 30 ASD children and compared to 30 TD controls in Egypt [27]. The authors found that ASD children typically had 75% fewer CD4^+^CD25^+^ T_regs_ compared with controls and that those with severe ASD had approximately 90% fewer CD4^+^CD25^+^ T_regs_. Two older studies also describe deficiencies in lymphocyte populations that may possess regulatory properties in ASD [39,40]. IL-35 is a recently described cytokine with inhibitory properties, produced primarily by regulatory T and B cells and is required for T_reg_-mediated immunosuppression. This may suggest that decreases in T_regs_ numbers or defects in T_regs_ function may drive lower IL-35, TGFβ1 and IL-10 levels. 

Exciting and intriguing data suggest that targeting the immune response by exerting greater control/regulation/suppression can be a beneficial therapy in ASD (reviewed in [41]), showing improvements of core-ASD and associated behaviors; including, social interactions, language, vocalization, stereotypic behavior, irritability, and emotional regulation. However, often these studies are limited to small trials that need careful replication. Cord blood cell transplantation was rich in T_regs_ and induced significant behavioral improvements in ASD [42,43]; although the specific cell type responsible for these positive outcomes has not yet been identified. Enhancing the power of T_regs_ to control responses can lead to better regulation. A more comprehensive understanding of the complex mechanisms of immune regulation in ASD will advance the development of targeted therapies.

## 5. Conclusions

To summarize, the results described here are in agreement with prior studies and suggest that regulatory anti-inflammatory mediators are decreased in ASD and could contribute to pathophysiology and behavioral symptoms. Furthermore, future studies, such as longitudinal measurements of the current subjects, could further elucidate the putative role of IL-35 concentrations as biological signatures of the progression and development of ASD. Additional studies will be necessary to better understand the relationship of IL-35 in ASD development and the role of this cytokine and its cognate receptors play in the brain.
